# Prevalence and Experience of Urinary Incontinence Among Elite Female Gaelic Sports Athletes

**DOI:** 10.1007/s00192-024-05893-2

**Published:** 2024-08-16

**Authors:** Elizabeth Culleton-Quinn, Kari Bø, Neil Fleming, Cinny Cusack, Déirdre Daly

**Affiliations:** 1 School of Medicine, Trinity College, Dublin, Ireland; 2 Department of Obstetrics and Gynecology, Norwegian School of Sports Sciences Oslo, Norway and Akershus University Hospital, Lørenskog, Norway; 3 Physiotherapy Department, Rotunda Hospital, Dublin, Ireland; 4 School of Nursing and Midwifery, Trinity College, Dublin, Ireland; 5 Discipline of Physiotherapy, Trinity Centre for Health Sciences, St James Hospital, James’s Street, Dublin, D08W9RT Ireland

**Keywords:** Experience, Female athletes, Prevalence, Urinary incontinence

## Abstract

**Introduction and Hypothesis:**

This study was aimed at identifying the prevalence and experience of urinary incontinence (UI) among elite female Gaelic sports athletes in Ireland.

**Methods:**

A cross-sectional study comprising an anonymous online survey of elite Gaelic sports (Camogie and Ladies Gaelic Football) players. Players were asked about their background information, knowledge of the pelvic floor and practice of pelvic floor muscle training (PFMT), as well as their experiences of UI. The prevalence and severity of UI was assessed using the International Consultation on Incontinence Questionnaire-UI Short Form (ICIQ-UI-SF) questionnaire. Logistic regression was used to evaluate risk factors.

**Results:**

A total of 185 players responded (25 ± 5 years) and 95.3% (*n* = 176) were nulliparous. Forty-one percent (*n* = 75) of players had learned about PFMT and 13% (*n* = 24) had performed PFMT within the last 4 weeks. The ICIQ-UI-SF was completed by 159 players, with UI reported by 61.6% (*n* = 98), 52% (*n* = 51) of whom experienced stress urinary incontinence (SUI). A significant association was found between UI and longer weekly sporting activity time (OR 1.05, 95% CI 1.002 to 1.008). Parous players were more likely to experience UI (*p* = 0.025). Jumping and sprinting activities were the most commonly reported triggers, with pre-voiding and wearing protection (liners, pads) the most commonly adopted UI management strategies. Only ten players reported receiving treatment for UI.

**Conclusions:**

Urinary incontinence is prevalent among this cohort of elite female Gaelic sports athletes. Many players used strategies to manage their UI whereas few sought help for what is a treatable condition. Findings suggest the need for education of players regarding pelvic floor health and treatment options available.

## Introduction

Pelvic floor dysfunction (PFD) is a collection of conditions, signs, and symptoms that can affect the pelvic floor with urinary incontinence (UI), which is defined as a “complaint of involuntary loss of urine,” the most commonly experienced PFD [1]. UI has been found to be more prevalent among athletes than among their non-athletic counterparts [2, 3]. Although the effect of physical activity and sport on the pelvic floor is not yet fully understood [4] research indicates that there are certain risk factors that may influence the prevalence of UI. Risk factors for UI include female sex, high-impact sport, long hours of training, and elite status as an athlete [5–8]. Stress urinary incontinence (SUI), which has been defined as “complaint of involuntary loss of urine on effort or physical exertion (e.g., sporting activities), or on sneezing or coughing” [1], appears to be the most commonly reported form of UI among female athletes [3, 6]. Research has indicated that athletes experience triggers for their UI, such as jumping activities, and adopt strategies, including pre-voiding, wearing pads, and fluid restriction, to try to manage and mitigate the symptoms of their UI in sport [6, 7, 9, 10]. A recent systematic review identified the need for sports-specific mixed methods or qualitative research to further examine the experience of PFD among elite sportswomen [9].

Gaelic team sports are the national and most commonly played sports in Ireland and include Camogie and Ladies Gaelic Football, where two 15-member teams (1 goalkeeper, 6 backs, 2 midfielders, and 6 forwards), playing on a large grass pitch (145 m by 90 m) aim to score points (over the bar) and goals (equal to three points, under the bar) on H-shaped posts during a 60-min game. These sports are also played in Australia, Britain, Europe, North America, the Asia/Gulf region, and Africa. Camogie is played with a hurley (stick) and a sliotar (small, hard, leather-covered ball) and players may strike the sliotar with the hurley, solo (move with the sliotar balanced or bouncing on the hurley), hand pass, or kick the sliotar [11]. The Camogie Association has a membership of over 600 Camogie clubs and over 100,000 players worldwide [12]. Ladies Gaelic Football is played with a round, Gaelic football (400 g) and players can kick, catch, solo (players drop the ball onto the foot and kick it back into their hands), bounce, or hand pass the ball [13]. The Ladies Gaelic Football Association has over 1,000 clubs with over 150,00 players worldwide [14]. Camogie and Ladies Gaelic Football players on county teams who compete in the respective National League and All-Ireland Championships represent the elite players in these sports.

To our knowledge, there has been no research to date investigating the prevalence and experience of UI among elite female Gaelic sports athletes. The study presented here is the quantitative component of a mixed-methods study investigating the prevalence and experiences of pelvic floor muscle dysfunction in elite female athletes/sportswomen that was aimed at investigating the prevalence and experience of UI and associated risk factors. A secondary objective was to investigate players’ knowledge of the pelvic floor muscles (PFMs) and pelvic floor muscle training (PFMT).

## Materials and Methods

### Study Population

This was a cross-sectional study involving elite female Gaelic sports athletes (county Camogie players and county Ladies Gaelic Football players) in Ireland. All players who fulfilled the inclusion/exclusion criteria, aged 18 years and over, and able to understand written English, were invited to participate in the study. Exclusion criteria were players who were aged 17 years and younger, pregnant or had given birth within the previous year or who had had previous pelvic surgery or pelvic disease.

### Survey Instrument Development and Evaluation

An online self-completed anonymous questionnaire was developed and administered using Qualtrics©. Background demographics and potential risk factors based on former studies in the area of UI generally and UI and sport were collected and included age, BMI, presence of medical risk factors for UI (including gynecological or bladder and bowel conditions, diabetes, asthma, lumbar spine, or pelvic girdle musculoskeletal symptoms), parity, types and amount of fluid intake (total fluids; total amount of bladder-irritant fluids: caffeinated, or carbonated drinks; proportion of bladder-irritant fluids/total fluids and alcohol consumption), and time spent in sporting activities per week [5, 7, 15–21]. The prevalence, nature, and severity of UI was assessed by the International Consultation on Incontinence Questionnaire-Urinary Incontinence Short Form (ICIQ-UI-SF) questionnaire, which has been shown to have reliability, validity, and sensitivity [22]. Players were classified as being continent if they answered “never” to the question “How often do you leak urine?” UI was further classified as stress urinary incontinence (SUI), urge urinary incontinence (UUI), mixed urinary incontinence (MUI) from answers to the options in the question “When does urine leak?” An Everyday Life (EDL) score (1–10) was obtained from a question “Overall how much does your leaking urine interfere with your daily life?” Finally, the ICIQ-UI-SF sum score indicating the severity of UI was calculated from the sum of the frequency, amount, and effect on daily life. Severity scores were categorized as slight (1–5), moderate (6–12), severe (13–18), and very severe (19–21).

The questionnaire also included questions about the players’ knowledge regarding the location of the PFMs and the practice of PFMT, their experience of UI (including triggers for UI and strategies employed to manage their UI) and whether players with UI had discussed or sought treatment for UI.

Validity and reliability were evaluated in October and November 2020 respectively, prior to commencing the study. Content validity was evaluated by 16 physiotherapists (14 women’s health physiotherapists, 1 in education and women’s health research, and 1 physiotherapist involved in the treatment of sports injuries in female Gaelic sports athletes) using a relevance rating scale [23]. The scale level content validity index average (S-CVI/average) was 0.997 and all 16 experts agreed on 96% of the items (46 out of 48). Reliability was tested using a convenience sample of club-level Camogie players, with 12 players completing the questionnaire on two occasions 1–2 weeks apart. Cohen’s Kappa, used to establish test–retest reliability, was Kappa range = 0.625 to 1.000, indicating good reliability.

A link to the questionnaire was emailed to the county players by the Gaelic Players Association (GPA), the player representative body for intercounty players in Ireland (~1,350 female players with ~1,280 aged 18 years or older) [24], which acted as gatekeeper for the study. The link was made available to the county Camogie players on two separate occasions: late November to mid-December 2020, for the end of the 2020 intercounty season, which had been delayed owing to the COVID-19 pandemic, and May to September 2021 to both county Camogie and Ladies Gaelic Football players for the 2021 intercounty season.

### Statistical Analyses

Data were exported to IBM SPSS version 26.0 statistics package and analyzed using descriptive statistics and logistic regression. Background characteristic variables were provided as numbers and percentages, means with standard deviation (SD), as appropriate, and the prevalence of UI was reported as frequency and percentage. Shapiro–Wilk and Kolmogorov–Smirnov tests were used to evaluate the normality of the continuous data. Chi-squared/Fisher’s exact test and the Mann–Whitney *U* test were used to evaluate differences between the two sports and to compare risk factors and background characteristics of players with and without UI. The confidence level was set as 95%, with significance at *p* < 0.05. Binary logistic regression analysis reporting odds ratios, 95% confidence intervals (CIs), and *p* values was carried out to evaluate risk factors for UI. The dependent variable was reporting UI.

## Results

### Background Characteristics of the Players

A total of 185 players (Camogie players *n* = 102, Ladies Gaelic Football players *n* = 83) submitted responses deemed sufficiently complete for analysis. Mean age (SD) of players was 25 (5) years [range 18 to 37 years]. Almost all players had never smoked or vaped (97.8%, 181 out of 185). Only 8 players were parous, with the majority being nulliparous (95.3%, 161 out of 169). Table 1 outlines the background characteristics of the players.

**Table 1:** Background characteristics of the players

Background characteristics^a, b^ (*n* =)^c^	All players	Camogie players	Ladies Gaelic football players	*p* value*ǂ
BMI, mean (SD) (*n* = 178)	22.9 (2.6)	23.4 (2.6) (*n* = 96)	22.4 (2.6) (*n* = 82)	p = 0.004*
Has a history of medical risk factor (*n* = 173)	83% (144/173)	80% (80/100)	87.7% (64/73)	p = 0.182**
Daily fluids^d^ (ml), mean (SD), (*n* = 185)	2,820.4 (901.1)	2,779 (996.3) (*n* = 102)	2,870.8 (771.1) (*n* = 83)	p = 0.311*
Daily bladder irritant^e^ fluids (ml), mean (SD), (*n* = 185)	807.8 (523.2)	727.8 (513.9) (*n* = 102)	906.0 (520.8) (*n* = 83)	p = 0.015*
Percentage of daily fluid intake as bladder irritants^e, f^, mean (SD), (*n* = 185)	29.5 (19.1)	26.3 (16.4) (*n* = 102)	33.4 (21.4) (*n* = 83)	p = 0.047*
Drinks alcohol (*n* = 184)	89.1% (164/184)	94.1% (95/101)	69/83 (83.13%)	p = 0.018**
Units alcohol/week, mean (SD), (*n* = 161)	5.2 (4.9)	4.4 (4.5) (*n* = 94)	6.3 (5.2) (*n* = 67)	p = 0.015*
Weekly time spent in sporting activity (min), mean (SD), (*n* = 126)	472.0 (175.8)	441.5 (156.2) (*n* = 70)	510.1 (192.3) (*n* = 56)	p = 0.040*

*BMI* Body Mass Index

To determine differences between sports, data were analyzed using *Mann–Whitney *U* test or **Chi-squared test

^a^Quantitative variables were expressed as means ± SD

^b^Categorical variables were expressed as numbers and percentage values

^c^Number of players who answered the question

^d^Non-alcoholic fluids

^e^Bladder-irritant fluids include caffeinated or carbonated drinks

^f^(ml of bladder-irritant fluids/ml of total fluids) × 100)

### Prevalence of Urinary Incontinence

A total of 159 players answered the ICIQ-UI-SF section of the questionnaire (Camogie players *n* = 79, Ladies Gaelic Football players *n* = 80). Ninety-eight players reported UI, giving an overall prevalence of 61.6%. The overall prevalence was slightly higher among the Ladies Gaelic Football players with 63.8% (51 out of 80) reporting UI and 59.5% (47 out of 79) of Camogie players reporting UI. However, there was no significant difference in the overall prevalence of UI between sports (Chi-squared = 0.305, df = 1, *p* = 0.581). Table 2 reports findings of the ICIQ-UI-SF, giving the prevalence of SUI, UUI, and MUI along with the EDL and ICIQ-UI-SF scores of the players reporting UI. There was no significance difference in prevalence of SUI, UUI, MUI, or the EDL or ICIQ-UI-SF scores between the two sports.

**Table 2:** Urinary incontinence findings from the International Consultation on Incontinence Questionnaire-UI Short Form (ICIQ-UI-SF) questionnaire

Descriptive	All players with UI (*n* = 98)	Camogie players with UI (*n* = 47)	Ladies Gaelic football players with UI (*n* = 51)	*p* value*, **
Prevalence of SUI only, % (*n*)	52% (51)	55.3 % (26)	49.0 % (25)	p = 0.533**
Prevalence of UUI only, % (*n*)	7.1 % (7)	10.6 % (5)	3.9 % (2)	p = 0.255***
Prevalence of MUI, % (*n*)	31.6 % (31)	29.8 % (14)	33.3 % (17)	p = 0.657**
Leaks for no obvious reason, % (*n*)	9.2 % (9)	4.2 % (2)	13.7 % (7)	p = 0.163***
EDL score, mean ± SD	2.3 ± 2.3	2.2 ± 2.2	2.3 ± 2.4	p = 0.862*
ICIQ-UI-SF sum score (mean ± SD)	6.1 ± 3.4	6.2 ± 3.7	6.0 ± 3.1	p = 0.732*

ICIQ-UI-SF, sum score indicated the severity of UI

To determine differences between sports, data were analyzed by *Mann–Whitney *U* test, **Chi-squared tests, or ***Fisher’s exact test

*EDL* Everyday Life Score, *SUI* stress urinary incontinence, *UUI* urge urinary incontinence, *MUI* mixed urinary incontinence

### UI Prevalence and Risk Factors

Significantly more of the parous players (100%, 8 out of 8) reported experiencing UI compared with the nulliparous players (60.3%, 85 out of 141; Fisher’s exact test *p* = 0.025). There was no significant difference in the prevalence of UI between those reporting medical risk factors (62.8%, 81 out of 129) compared with those with no medical risk factors (54.6%, 12 out of 22; Chi-squared = 0.540, df = 1, *p* = 0.462). Shapiro–Wilks and Kolmogorov–Smirnov tests on the continuous variables of age, BMI, fluid intake, alcohol consumption, and time spent in sporting activity per week indicated non-normal distributions. Table 3 depicts the evaluation of the effect of these risk factors on the prevalence of UI.

**Table 3:** Comparison of risk factors between players with and without UI

	No UI, *n* (mean rank)	UI, *n* (mean rank)	Mann–Whitney *U* test results
Age (*n* = 159)	61 (80.6)	98 (79.7)	*U* = 2,954.500
			*z* = −0.123
			*p* = 0.902
BMI (*n* = 153)	57 (83.3)	96 (73.2)	*U* = 2,375.000
			*z* = −1.363
			*p* = 0.173
Daily fluid intake (*n* = 159)	61 (80.3)	98 (79.8)	*U* = 2968.500
			*z* =—0.073
			*p* = 0.942
Daily amount^a^ bladder irritants (*n* = 159)	61 (67.8)	98 (87.6)	*U* = 3,730.500
			*z* = 2.634
			**p = 0.008**^c^
Percentage of daily fluid intake as ^a^ bladder irritants^b^ (*n* = 159)	61 (67.0)	98 (88.1)	*U* = 3,784.000
			*z* = 2.818
			**p = 0.005**^c^
Weekly units of alcohol (*n* = 136)	48 (59.9)	88 (73.2)	*U* = 2,524.500
			*z* = −1.888
			*p* = 0.059
Minutes of sporting activity (*n* = 125)	45 (45.7)	80 (72.7)	*U* = 2,577.000
			*z* = 4.000
			**p < 0.001**^c^

BMI Body Mass Index

^a^Bladder-irritant fluids include caffeinated or carbonated drinks

^b^(ml of bladder-irritant fluids/ml of total fluids) × 100)

^c^Reject null hypothesis

Players who experienced UI were found to drink greater daily amounts of bladder irritants or drank a higher percentage of daily fluids as bladder-irritant fluids ((ml of bladder-irritant fluids/ml of total fluids) × 100). Similarly, those players experiencing UI reported longer average weekly minutes of sporting activity than players who reported that they were continent. Although weekly units of alcohol did not appear to be higher in those players reporting UI, this finding approached significance and was therefore included in the logistic regression model along with the above findings.

Results from logistic regression analysis indicated that players with a greater number of weekly minutes of sporting activity were more likely to experience UI (OR 1.05, 95% CI 1.002–1.008).

### Players’ Experience of Urinary Incontinence (Triggers for UI, Strategies to Manage UI)

**Fig. 1:**
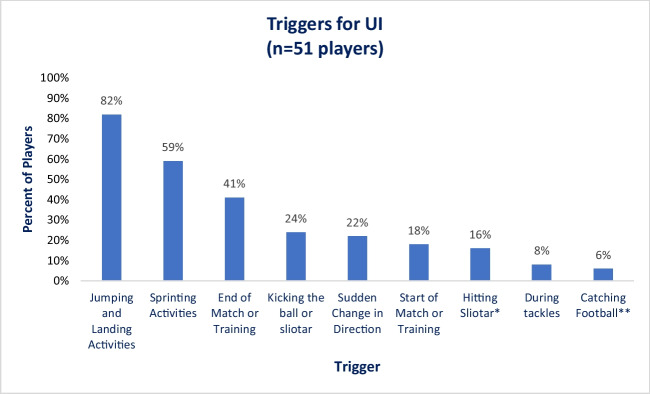
Triggers for urinary incontinence (UI). *Sliotar refers to the small hard leather-covered ball used in Camogie; **Football refers to the round football used in Ladies Gaelic Football

Of the 98 players reporting UI, 78 answered the question as to whether they noticed leakage at particular times during their sporting activity. Of these, 65% (51 out of 78) reported triggers for their UI (Fig. 1).

**Fig. 2:**
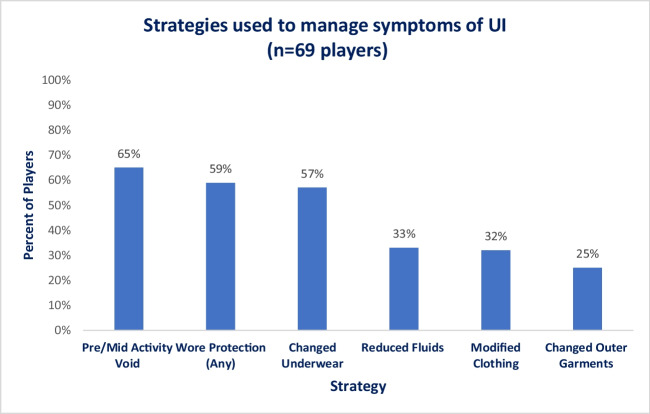
Strategies used by players to manage symptoms of urinary incontinence (UI)

Eighty players answered questions regarding the use of strategies to manage or mitigate their UI, with 86% (69 out of 80) reporting that they used strategies to manage their UI during their sporting activity (Fig. 2).

Seventy-eight players answered the questions asking if they had talked to anyone about their UI, with 24% (19 out of 78) of reporting that they had talked to another person about their UI. Most commonly, players spoke to family (68%, 13 out of 19) or friends (58%, 11 out of 19); 2 players (11%, 2 out of 19) had spoken to a physiotherapist, and 2 players (11%, 2 out of 19) had spoken to a GP about their UI.

Eighty of the 98 players with UI answered the questions regarding receiving treatment of their UI, with only 13% (10 out of 80) reporting that they had received treatment. All of the Ladies Gaelic Football players with UI answered this question, with only 8% (4 out of 51) receiving treatment. Twenty-nine Camogie players answered this question, with 21% (6 out of 29) reporting that they had received treatment. All 8 parous players answered this question, with half of these players reporting that they had received treatment for their UI.

Eight of the ten players received treatment for their UI from a physiotherapist, 6 from a gynecologist, 3 from a GP, and 1 from a nephrologist. Mostly commonly, the players reported PFMT as the recommended treatment, with 8 prescribed PFM strengthening exercises and 6 prescribed pelvic floor relaxation exercises or stretches. Two players were prescribed bladder training and 2 were prescribed medication to treat their UI.

### Knowledge and Practice of Pelvic Floor Muscles and Pelvic Floor Muscle Training

**Fig. 3:**
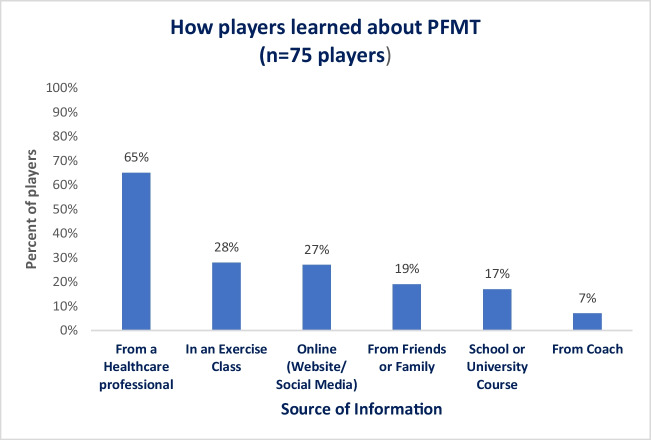
How players learned about pelvic floor muscle training (PFMT)

All players (*N* = 185) answered the questions concerning the pelvic floor muscles (PFMs) and pelvic floor muscle training (PFMT). Fifty-five percent (101 out of 185) said that they knew where the PFMs are, 41% (75 out of 185) said that they had learned about PFMT, 26% (48 out of 185) said that they were confident in performing PFMT, and only 13% (24 out of 185) reported that they had done PFMT in the last 4 weeks. Figure 3 identifies how players learned about PFMT.

## Discussion

This quantitative study is to our knowledge the first to investigate the prevalence and experience of UI among elite female Gaelic sports athletes (Camogie and Ladies Gaelic Football players) in Ireland. The differences between the Camogie and Ladies Gaelic Football players regarding age, BMI, parity, presence of medical risk factors, and total fluid intake were relatively small and considered to be of no clinical significance. Camogie players were found to have a higher consumption of weekly units of alcohol and the Ladies Gaelic Football players were found to have a higher consumption of bladder-irritant fluids and interestingly, the Ladies Gaelic Football players reported significantly longer weekly sporting activity time (*p* = 0.04), which was identified as a significant risk factor for UI in this current study.

### Prevalence of UI

Although the prevalence of UI was slightly higher among the Ladies Gaelic Football players than among the Camogie players, there was no significant difference between sports in the prevalence of UI, the prevalence of SUI, UUI, MUI, or the EDL or ICIQ-UI-SF scores.

The overall prevalence of UI among these elite players was high (61.6%). This may not be surprising owing to the high velocity, high impact, and physical nature of the two sports, which include kicking a large ball (Ladies Gaelic Football) or striking a sliotar (Camogie), as well as running, sprinting, jumping, catching, and passing over a large 145 m × 90 m pitch. Research has indicated that high-impact sports, elite status, and long hours of training are risk factors for UI [3, 6, 7, 25], which is supported by the findings of the current study, with logistic regression indicating that players who had longer weekly sporting activity time were significantly more likely to experience UI.

Rugby is a high-impact team sport played on a large grass pitch and a study by Sandwith and Robert involving Canadian Intervarsity female rugby players found that over half (54%) reported UI [20]. A recent study by McCarthy-Ryan et al. investigating the prevalence of UI among female rugby players in the UK and Ireland, who were playing rugby at any level, reported a rugby-related prevalence of SUI of 43% [26]. Pires et al., in a review evaluating the prevalence of UI and the influence of sport modality, reported that the prevalence of UI in high-impact sports (involving running and jumping) ranged from 14.3% to 75.6%. Of the team sports, volleyball (75.6%) was found to have the highest prevalence, followed by indoor football (50.0%) [8]. Gaelic sports involve less jumping than volleyball, but more jumping than rugby and indoor football, and this could potentially explain the relative prevalence of UI reported here.

Stress urinary incontinence (52.0%) was the most common form of UI reported in this study, and almost one-third reported MUI, with few (7.1%) reporting UUI only. Of those players who reported triggers for their UI, over four-fifths (42 out of 51) reported jumping activities and over half (30 out of 51) reported sprinting activities to be triggers. This is consistent with the findings of previous research reporting that SUI is the most common form of UI among high-impact athletes and is considered to be a consequence of the increased abdominal pressure experienced and the impact of ground reaction forces on the pelvic floor during high-impact sport [6, 8, 25]. In addition, around two-fifths of the players reporting triggers said that UI occurred toward the end of matches or training, potentially suggesting pelvic floor fatigue [21].

Although not found to be statistically significant in the regression analysis model, the results suggested that players who included more bladder irritants in their fluid intake were more likely to experience UI, which corresponds with findings from previous research literature [16, 17]. This suggests a need for education regarding bladder health and type of fluids among the players.

Parity appeared to influence the prevalence of UI, with all eight parous players experiencing UI. Four parous players sought treatment, although it is unknown if these players sought treatment before or after returning to their sport. Groom et al. recently published a return to running after pregnancy protocol that highlighted the fact that postpartum women benefit from individualized pelvic floor assessment and rehabilitation for the prevention and management of PFD on their return [27]. A recent qualitative study by Davenport et al. suggested that there appears to be limited support for elite female athletes in their return to sport postpartum and that there is a need for evidence-based return-to-sport protocols [28]. Interestingly, half of the parous players did not receive treatment for their UI. Future research should include surveying or interviewing past county players to explore if PFD and UI post-childbirth was a factor in leaving elite-level play and if they were aware of the treatment options available. Consideration should be given to the development of a protocol for a return to Gaelic sport postpartum following further mixed-methods research conducted with these elite players.

### Knowledge of PFMs/PFMT and Experience of UI (Impact, Triggers, Strategies, and Treatment)

Although many players knew where the PFMs are, and 41% had learned about PFMT, not all were confident in performing the exercises and few players had done PFMT within the last 4 weeks. UI is a treatable condition. However, only 10 players reported receiving treatment for their UI, and this was most commonly from a physiotherapist and mainly in the form of PFMT. A Cochrane review found that PFMT can improve or cure UI and is most effective in the treatment of SUI [29]. Similar to the findings of previous research, these data suggest a need for education around PFMT and treatment in these elite players [3, 6–9]. In addition, future research should consider evaluating the effect of PFMT in elite Gaelic sports athletes.

The mean (SD) ICIQ-UI-SF severity score of 6.13 (3.37) was within the moderate range (6 to 12 out of 21) [30]. Despite the high prevalence and moderate severity, the impact of UI on the players’ everyday life was relatively low (mean (SD) EDL score/10 of 2.29 (2.28)), which may be expected in a predominantly nulliparous group of relatively young players.

Few players spoke to another person about their UI and most commonly spoke to family or friends. Although few discussed their UI or sought help for what is a treatable condition, many players reported using strategies to try to manage and mitigate their symptoms. The strategies most commonly used were pre-voiding, wearing protection, modifying or changing clothing during sports, or reducing fluids, the latter of which is of concern owing to the need for appropriate hydration in athletes. Once again, this suggests a need for education regarding UI/PFD and treatment options available and is consistent with the findings of previous research [6, 7, 9].

### Strengths and Limitations

The strengths of this study include the fact that the sports included have not been investigated to date and, although the questions exploring players’ experiences were quantitative in nature, the responses helped to inform the interview guide for the qualitative component of the larger mixed-methods study. Limitations include the relatively low response rate from the overall number of Gaelic players. It is also acknowledged that players who were experiencing UI may have been more likely to respond than players who were continent. Therefore, findings regarding the prevalence of UI and associated risk factors must be interpreted with caution and viewed as prevalence amongst this cohort of female players. However, having a total of 185 responses from these elite female athletes who were playing two different sports adds to the international literature about sportswomen’s experience of UI and new knowledge in Ireland. In addition, the prevalence reported here is similar to that of other high-impact, field-based sports played on a large grass pitch, such as rugby [20].

This study involved female Gaelic sports athletes who were 18 years and older, predominantly nulliparous, playing at an elite level, with long hours of training, and therefore the results may not be generalizable to club-level Camogie and Ladies Gaelic Football players, or to other sports. Further research into the prevalence and experience of UI could include club-level and adolescent female Gaelic sports players.

## Conclusions

Urinary incontinence is prevalent among this cohort of elite female Gaelic sports athletes. Risk factors for UI among the players include parity and greater weekly time spent in sporting activity. Players utilized strategies to manage the symptoms of UI rather than seek help for what is a treatable condition. This study suggests a need for increased awareness and education regarding pelvic floor health and the treatment of UI among these players.

## Data Availability

Data is available from the author on request.
